# You do you: susceptibility of temporal binding to self-relevance

**DOI:** 10.1007/s00426-023-01906-9

**Published:** 2024-01-03

**Authors:** Felicitas V. Muth, Sophia Ebert, Wilfried Kunde

**Affiliations:** Department of Psychology, Julius-Maximilians-University of Würzburg, Röntgenring 11, 97070 Würzburg, Germany

## Abstract

The self-prioritization effect suggests that self-relevant information has a processing advantage over information that is not directly associated with the self. In consequence, reaction times are faster and accuracy rates higher when reacting to self-associated stimuli rather than to other-related stimuli (Sui et al., Journal of Experimental Psychology: Human Perception and Performance 38:1105–1117, 2012). This spurs the assumption that self-associated action–effects should also be perceived earlier than other-related outcomes. One way to measure this is temporal binding. Previous research indeed showed that the perceived temporal interval between actions and self-associated outcomes was reduced compared to friend- and other-associated outcomes. However, the employed method (interval estimations) and several experimental design choices make it impossible to discern whether the perceived shortening of the interval between a keypress and a self-relevant outcome is due to a perceptual shift of the action or of the action–effect or both. Thus, we conducted four experiments to assess whether temporal binding can indeed be modulated by self-relevance and if so where this perceptual bias is located. The results did not support stronger temporal binding for self- vs other-related action–effects. We discuss these results against the backdrop of the attentional basis of self-prioritization and propose directions for future research.

**Supplementary Information:**

The online version contains supplementary material available at 10.1007/s00426-023-01906-9.

## Introduction

When humans voluntarily act to cause a change in their environment and the intended change eventually presents itself a sense of agency for the action as well as the outcome arises (Haggard & Tsakiris, 2009). Studies have shown that the interval between such voluntary actions and subsequent action–effects is perceived as shortened compared to identical intervals caused by involuntary movements or third parties (Buehner & Humphreys, 2009; Haggard et al., 2002). This perceived shortening of the interval is referred to as temporal binding or intentional binding (for a review see Moore & Obhi, 2012). There is good reason to believe that outcomes which the agent has intended and therefore predicted are perceived somewhat differently than randomly occurring events in the environment. Consequently, temporal binding is widely employed as an implicit measure for sense of agency even though it is debated whether it is fair to do so (for a critical review see Buehner, 2012; Kirsch et al., 2019; Thanopoulos et al., 2018). Temporal binding is susceptible to various factors such as valence (Christensen et al., 2016; Moreton et al., 2017; Takahata et al., 2012) and control (Beck et al., 2017) just to name a few and relates to the sense of agency a person has in a specific situation. Other strands of research have focused on the origin of temporal binding as either a phenomenon of mere causality or as product of multisensory integration (Hoerl et al., 2020; Klaffehn et al., 2021). Finally, all this research converges on the observation that the interval between an action and a causally linked sensory event is perceived as shortened in comparison to identical intervals lacking causal or intentional links. Up-close, the perceived shortening is typically comprised of a forward shift of the action towards the sensory event and a backward shift of the sensory event towards the preceding action (for a discussion of time awareness of sensory events see Tsakiris & Haggard, 2005).

While the self and self-conception are at the center of the sense of agency, research in the field has mainly concentrated on the self-relevance of the action, i.e., whether an action was voluntary or involuntary (Haggard & Clark, 2003), whether it was freely chosen or forced (Caspar et al., 2018), and whether it was executed by the actor or not (Pfister et al., 2014). Other studies analyzed the effect of joint actions on the sense of agency in human–human pairings or in human–machine interactions (for a review on social agency, see Silver et al., 2021). Surprisingly, thus far little research has been published on the self-relevance of action–effects.

Self-relevant information is processed faster and reactions to self-relevant stimuli are less error-prone than reactions to stimuli which have not been associated with the self (Sui et al., 2012). In experiments probing the self-prioritization effect, participants are asked, mostly by instruction, to associate a random geometric shape with themselves, another one with a friend, and a third one with a stranger. Afterwards, they complete a classification task in which participants see a shape label pairing and have to decide whether shape and label match. Typically, responses in self-match trials are fastest and least error-prone (Sui et al., 2012). Self-prioritization has also been found for arbitrary ownership (Constable et al., 2019; Cunningham et al., 2008) in combination with valence and reward (Golubickis et al., 2021; Sui & Humphreys, 2015). It also extends to other outcome domains such as auditory and tactile stimuli (Schäfer et al., 2016) as well as generalized concepts, i.e., a music instrument presented visually or auditory, or a shape with varying characteristics (Schäfer et al., 2015). Self-related objects are not only processed faster but also perceived to be more valuable (Kahneman et al., 1991). Thus, Humphreys and Sui (2016) proposed a neural network of personal significance in which areas for self-referential processing interact with areas of attentional control (see Sui et al., 2013). Self-relevance speeds up the focusing of attention during decision making such that when self-relevant information is processed, the attentional spotlight narrows in on them a lot faster than when a target is not self-related (Golubickis & Macrae, 2021b). Self-prioritization does, however, not only reside on a central stage influencing attention and action selection, rather it also influences movement production and execution (Constable et al., 2011; Desebrock & Spence, 2021; Desebrock et al., 2018). Consequently, it seems plausible to assume that actions involving a higher degree of agency should, conversely, also be conceived as more self-relevant than simple automated acts (Wegner, 2002). But is this link bidirectional? Does a higher degree of self-relevance also lead to a stronger sense of agency?

Makwana and Srinivasan (2019) were the first to address this question with an interval estimation task. Participants’ keypresses produced either stimuli which were previously associated with the self or a friend or a stranger. Subsequently, they were asked to estimate the duration of the interval between the keypress and the appearance of one of the three stimuli. Results showed that temporal binding was stronger, i.e., the interval perceived as shorter, when participants produced stimuli associated with the self as compared to stimuli associated with a friend or a stranger. Chiarella et al. (2020) extended these findings and suggest that promoting self-other connections e.g., through meditation, eliminates advantages of self-referential processing in postdictive temporal binding (binding caused by observation of a just encountered action–effect episode) but not the early process of self-prioritization.

One crucial shortcoming of the employed method is that it is impossible to discern whether the perceived shortening of the interval stems from a perceptual shift of the action towards the action–effect or a shift of the action–effect towards the action or a reciprocal attraction. Temporal binding is comprised of action binding (perceived later point in time of an action that produces an action–effect compared to an action that does not) and effect binding (perceived earlier point in time of an event that was produced by an action compared to an event that was not). Action binding and effect binding might be shaped by different processes (Hon, 2023; Tanaka et al., 2019) and increases in one component can be associated with decreases in the other (Lush et al., 2019; Wolpe et al., 2013; Yamamoto, 2020). Empirically, action binding and effect binding are uncorrelated across participants (Tonn et al., (2021) calling for separate analyses of the two rather than a composite measure like interval estimation.

Regarding self-relevance, there is reason to speculate that self-relevant stimuli impact effect binding and action binding differently. For example, if self-relevant stimuli get more attention than other-related stimuli they are perhaps accessible earlier to the system, thereby prompting stronger effect binding. Alternatively, devoting attention to a stimulus likely also increases reliability (or conversely reduces noise) of processing these stimuli, including processing of the stimulus’ timepoint. Action binding and effect binding have been shown to reflect the reliability processing differences of these two events, with larger binding effects for the relatively less reliable event (Klaffehn et al., 2021). Thus, self-relevant stimuli might be processed with higher levels of reliability than other-relevant stimuli, whereby action binding might increase.

To conclude there are methodical and theoretical reasons to have a look at the impact of self-relevance using a different measure, i.e., the Libet clock, which is what the present study intended to do.

## Experiment 1: self-relevant shapes

### Methods

All experiments presented here were preregistered on the Open Science Framework (OSF) and raw data as well as additional material is available (https://osf.io/pq43j/). The study was approved by the ethics committee of the psychology department of the Julius-Maximilians-University of Würzburg (GZEK 2021-63).

#### Participants

Sixteen participants (2 m, 14 f; 13 right-handed, 1 left-handed, 2 ambidexter) ranging between 22 and 64 in age (*M* = 31.9, SD = 12.4) were recruited over the university’s study platform SONA. Based on the large effect (*d* = 0.95) of self-relevance on temporal binding reported by Makwana and Srinivasan (2019), a sample of 13 should have sufficed to detect the effect (α = 0.05; power = 0.86). However, previous studies using the Libet clock to measure temporal binding showed smaller effect sizes. For example, Ruess et al. (2017) reported an effect of *d* = 0.65 for action binding. Thus, we based our targeted sample size on an a priori sample size calculation using G*Power (Faul et al., 2009) with a more conservative effect estimate of *d* = 0.65, α = 0.05 and a power of 0.85 resulting in 24 participants. Of these, 8 had to be excluded due to exclusion criteria preregistered on OSF. All participants were naïve to the study purpose and provided informed consent prior to the study. They received monetary compensation for their voluntary participation. Seven participants had to be excluded due to high error rates (> 25%) in the temporal binding task when asked to name the shape presented. Another participant was excluded because they did not remember their self-associated shape correctly after the temporal binding task.

#### Stimuli and task procedure

**Fig. 1:**
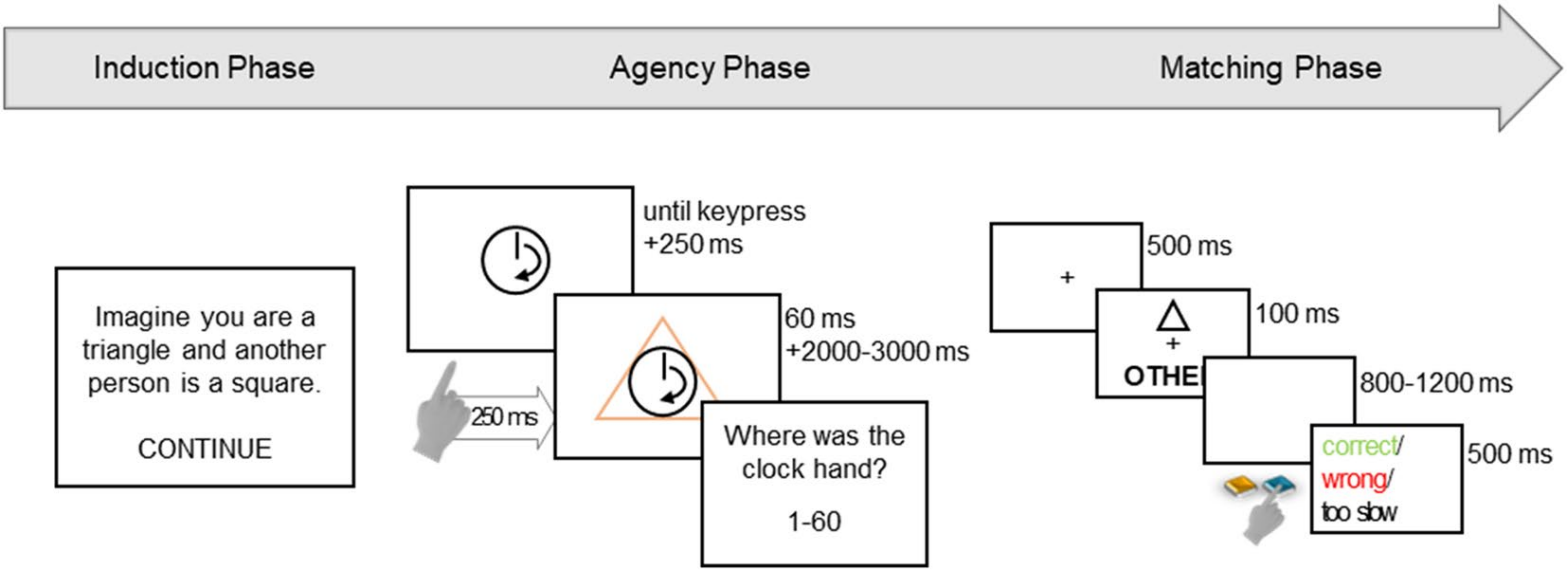
Trial procedure in Experiment 1. Note. Participants started with a short induction phase where they were assigned a geometric shape, while another shape was assigned to another person. Subsequently, participants performed the temporal binding task in which they pressed the space bar to randomly produce one of the two shapes. At the end of each trial, they were asked to report the position of the clock hand at either their keypress or the appearance of the shape. Finally, there was a matching task in which participants were presented a shape label pairing and were to decide whether it was a match or not

The experiment was run on stationary lab computers connected to LCD monitors with a screen size of 21.5ʺ (resolution 1920 × 1080 px) and a refresh rate of 60 Hz. It consisted of four phases: induction phase, agency phase, matching phase, and questionnaires (see Fig. 1).

#### Induction phase

At the beginning of the experiment, participants were assigned one of two shapes (square or triangle), the shape was counterbalanced across participants. In a short induction phase, they were asked to identify themselves with the respective shape while the other shape was said to represent someone else. The information (“Imagine you are a triangle/square and another person is a square/triangle”) was provided in German as text on the screen as well as over the computer’s speakers. In addition, during this induction phase and the subsequent agency phase the mouse cursor was shaped according to the own shape for participants to encounter control over the self-relevant shape.

#### Agency phase

Following the induction phase, participants completed the agency phase during which they performed a temporal binding task. A clock face of 240 px diameter with a rotating clock hand was presented at the center of the screen and voluntary keypresses resulted in the presentation of either the self-associated or the other-associated shape in the middle of the clock. Subsequently, participants reported the position of the clock hand at either the time of the keypress or the time of the appearance of the shape. This report was given by means of the keyboard (0–60) in correspondence with the minutes on a clock face. A full rotation of the clock lasted 2500 ms and participants were asked not to press a key within the first half revolution. The starting position of the clock hand was selected randomly. In the agency phase, participants encountered four different conditions:

1. *Action operant*—In this condition, participants pressed the spacebar to produce a geometric shape which was presented around the clockface. Once participants had pressed the spacebar, one randomly selected geometric shape appeared after a delay of 250 ms and was displayed for 60 ms (see also Haggard et al., 2002; Ruess et al., 2017). Afterwards the clock hand continued rotating for 2000–3000 ms. Then, participants were asked for the location of the clock hand at the time of their keypress (“Where was the clock hand when you pressed the key (0–60)?”). Sizes of the geometric shapes were determined to cover similar areas, that is, the square measured 384 × 384 px and the triangles base was 540 px wide and it was 540 px high. Both geometric shapes as well as the clockface were centered at the middle of the screen.
2. *Effect operant*—This condition was equal to the action operant condition. However, this time, participants were asked for the clock hand position at the time of the action–effect (“Where was the clock hand when the shape appeared?”).
3. *Action baseline*—In this condition, participants pressed the spacebar, but no geometric shape was displayed. After the keypress, the clock hand continued rotating for a random delay between 2000 and 3000 ms. Subsequently, participants indicated the position of the clock hand at the time of the keypress.
4. *Effect baseline*—In this condition, participants were asked to refrain from keypresses. Instead, after a random delay of 1250–3750 ms one of the two shapes appeared on the screen for 60 ms. After the clock hand had continued rotating for an additional 2000–3000 ms, participants indicated the position of the clock hand when the shape appeared.

In every block except for the action baseline bock, each geometric shape was presented 27 times resulting in a total block length of 54 trials. When participants committed an error in a trial, i.e., pressed the spacebar too early or at all in the effect baseline condition, or gave an estimation greater than 60, the trial was discarded, reshuffled to a later position in the block, and error feedback displayed. Between blocks, participants could take short breaks. The specific instruction as well as the event (action or action–effect) to be attended were given at the beginning of each block. In addition to the four working blocks, there was a practice block consisting of ten unbroken trials of the first condition. The order in which participants encountered the conditions was counterbalanced across participants, however, both baseline and both operant blocks were always executed after one another. Thus, there were four different possible presentation orders of the conditions. To check whether participants did pay attention to the shape in all conditions, they were asked to indicate what shape they had just seen after every fourth trial. These attention checks were executed with the mouse cursor. Performance in these attention checks was used as exclusion criterion (see preregistration [https://osf.io/pq43j/registrations] for details).

#### Matching phase

In the third part of the experiment, participants completed a matching task as in Sui et al. (2012). The task was to decide whether a presented shape and label were matching according to the association learned in the induction phase. Participants responded with the *S* and *L* key to indicate matches or no-matches. Mapping of the keys was counterbalanced across participants and match/no-match responses. Trials started with the presentation of a fixation cross in the middle of the screen for 500 ms. Subsequently, one of the two shapes (square, triangle) that were 90 px wide and 90 px high and either of the labels (self = “O O O I C H O O O”; other = “O O A N D E R E O O”) were presented above and below the fixation cross for 100 ms. Afterwards the screen turned white again until participants pressed a key or 1200 ms had passed. Finally, participants received feedback (correct, wrong, too slow) for 500 ms. Participants completed 110 trials in the matching task (25 matching and 25 non-matching trials per shape association; see also Frings & Wentura, 2014) of which the first 10 were considered practice trials and thus not included in the analysis.

#### Questionnaires

Finally, participants filled out German versions of the NPI-13 (Brailovskaia et al., 2019) as well as the PHQ-9 (Gräfe et al., 2004). We will not discuss the results of the questionnaires in this research as they were collected and used for a master’s thesis.

#### Data analysis

To analyze temporal binding, we first calculated participants’ estimation errors in each trial as the difference between the participants’ estimation and the actual timing of the event to be judged. Subsequently, two 2 × 2 analyses of variance (ANOVA) with condition (baseline vs. operant) and relevance (self vs. other) as within-subjects factors and the estimation error as dependent variable were conducted for actions and action–effects separately. Follow-up analyses were conducted via two-tailed, paired *t*-tests. The difference between estimation errors in the operant conditions and the respective baseline conditions is referred to as action binding and effect binding respectively. Positive values indicate that events were judged to have happened later in the operant compared to the baseline condition while negative values represent a shift to an earlier point in time. Finally, to illustrate the evidence, Bayesian analyses were performed in JASP (Dienes, 2014; JASP Team, 2018, Version 0.14). Based on the effects of action binding and effect binding reported for predictable delays of 250 ms by Ruess et al. (2017) and a lack of evidence that the effect size would be small relative to the maximal plausible effect size (Dienes, 2019), we modeled *H*_1_ for action binding as normal distribution with a scale factor of 14.07 ms (*BF*_N(0,14.07 ms)_). For effect binding, we modeled *H*_1_ as normal distribution with a standard deviation of 53.68 ms (*BF*_N(0,53.68 ms)_). As there is insufficient information on the size of the effect of self-relevance on temporal binding, the default Cauchy prior of 0.707 was used for those Bayesian *t*-tests. This means that we were 50% confident that the true effect size lies between − 0.707 and 0.707 which is common in social sciences (Bartlett, 2017).

As the self-prioritization effect is typically reported as the difference in RTs or error rates between self-match and other-match trials (e.g., Schäfer et al., 2021), we calculated mean reaction times and error rates for each trial type individually. Subsequently, we conducted two-tailed, paired *t*-tests. Effect sizes for all paired *t* tests were calculated as *d*_z_ = $\frac{t}{\sqrt{n}}$. Here again, we used Bayesian analyses to illustrate the evidence for any effects using the default Cauchy prior of 0.707 in JASP (JASP Team, 2018). In addition, in Table 2, we report *d*’ as a measure of general task performance. Analyses including no-match trials can be found in Table 3 in the Supplement.

### Results

**Fig. 2:**
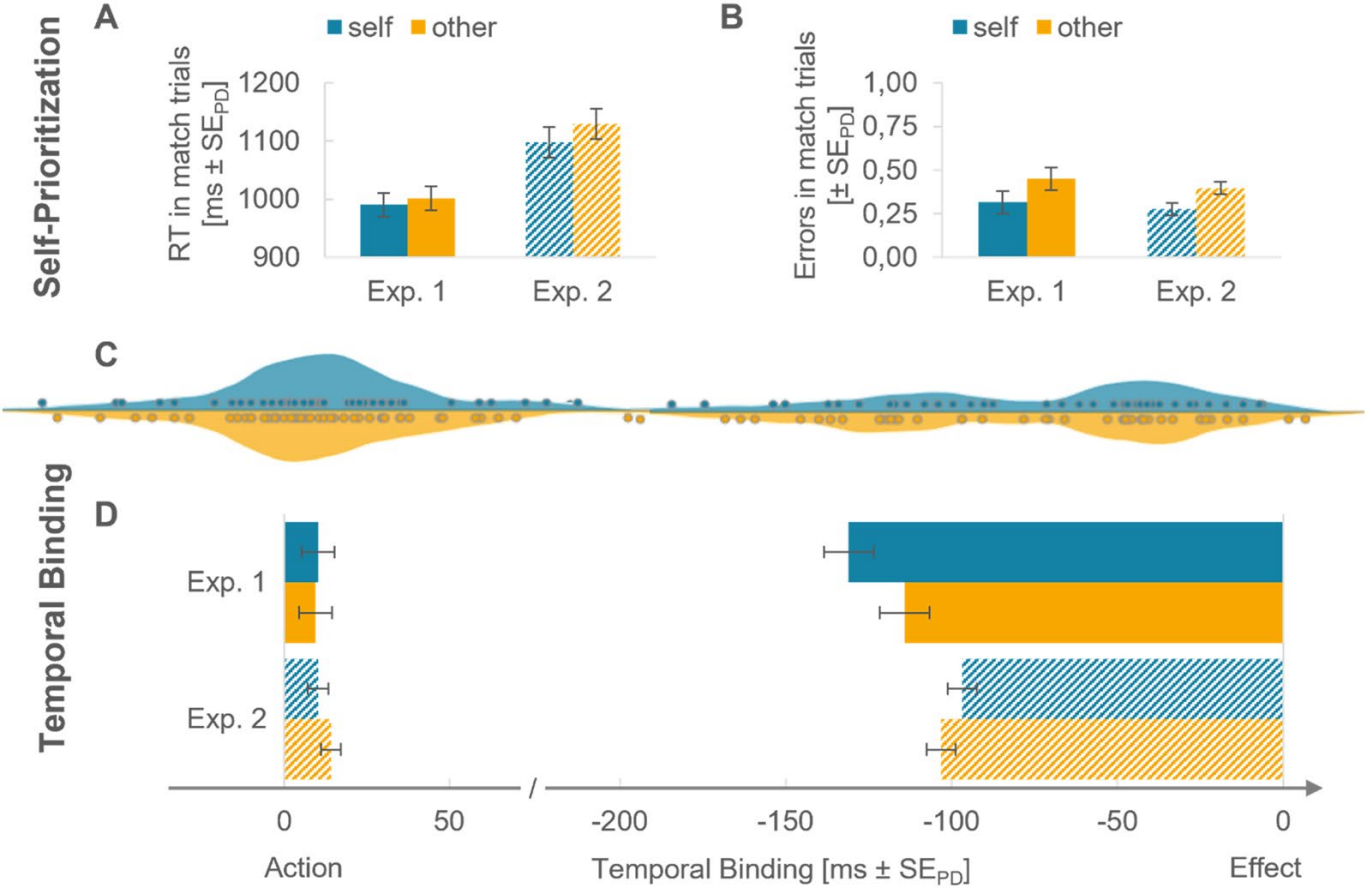
Self-prioritization effect and temporal binding in Experiments 1 and 2 using visual action–effects. Note. Stimulus association is color-coded: blue is self-related, orange is other-related. **A** Mean reaction times in self-related and other-related matching trials separately for Experiment 1 and Experiment 2. Error bars in all panels depict standard errors for paired differences. **B** Mean error rate in self-related and other-related matching trials separately for Experiment 1 and Experiment 2. **C** Distribution of action binding and effect binding combined for Experiments 1 and 2. Individual points represent participants. **D** Temporal binding in ms separately for Experiment 1 and Experiment 2. Bars from left to right depict the action shift in the operant relative to the baseline condition, i.e., action binding. Bars from right to left show the outcome shift in the operant relative to the baseline condition, i.e., effect binding

When reporting the results, we start with the matching task, i.e., the self-prioritization effect as this served as manipulation check to control whether participants assigned different weights to the self-related compared to the other-related stimulus. Subsequently, we present the data of the temporal binding task (see Fig. 2D).

#### Self-prioritization

Participants only showed a trend towards self-prioritization in the error rates but not in reaction times. Thus, the results must be interpreted with caution. Error rates were descriptively but not statistically lower in self-match trials compared to other-match trials, *t*(15) = 2.09, *p* = 0.054, *d*_*z*_ = 0.52, *BF*_10_ = 1.422, ∆ = − 13.5%. There was moderate evidence that reactions times did not differ significantly between self-related and other-related trials, *t*(15) < 1, *BF*_10_ = 0.290, ∆ = − 11.0 ms (see Fig. 2A, B).

#### Temporal binding

The 2 × 2 ANOVA for action binding with condition (baseline vs. operant) and relevance (self vs. other) did not reveal any significant main effect or interaction, main effect condition *F*(1,15) = 1.60, *p* = 0.225, ${\eta }_{p}^{2}$ = 0.10, *BF*_10_ = 1.00, all other *F*s < 1. As such, there was no evidence for either action as perceived to have happened later when they were followed by a shape as compared to happening in isolation.

Contrary, the 2 × 2 ANOVA for effect binding with condition (baseline vs. operant) and relevance (self vs. other) revealed a main effect of condition, *F*(1,15) = 23.16, *p* < 0.001, ${\eta }_{p}^{2}$ = 0.61, *BF*_10_ = 216.69, as well as a condition × relevance interaction, *F*(1,15) = 4.98, *p* < 0.041, ${\eta }_{p}^{2}$ = 0.25. Both outcomes were perceived to have happened earlier when they were preceded by a keypress as compared to happening in isolation. In addition, self-related outcomes resulted in stronger effect binding compared to other-related outcomes, *t*(15) = 2.23, *p* = 0.041, *d*_*z*_ = 0.56, ∆ = 16.9 ms. The main effect of relevance was not significant, *F*(1,15) < 1. Bayes factors for all comparisons between self-related and other-related action binding and effect binding are listed in Table 1.

**Table 1:**
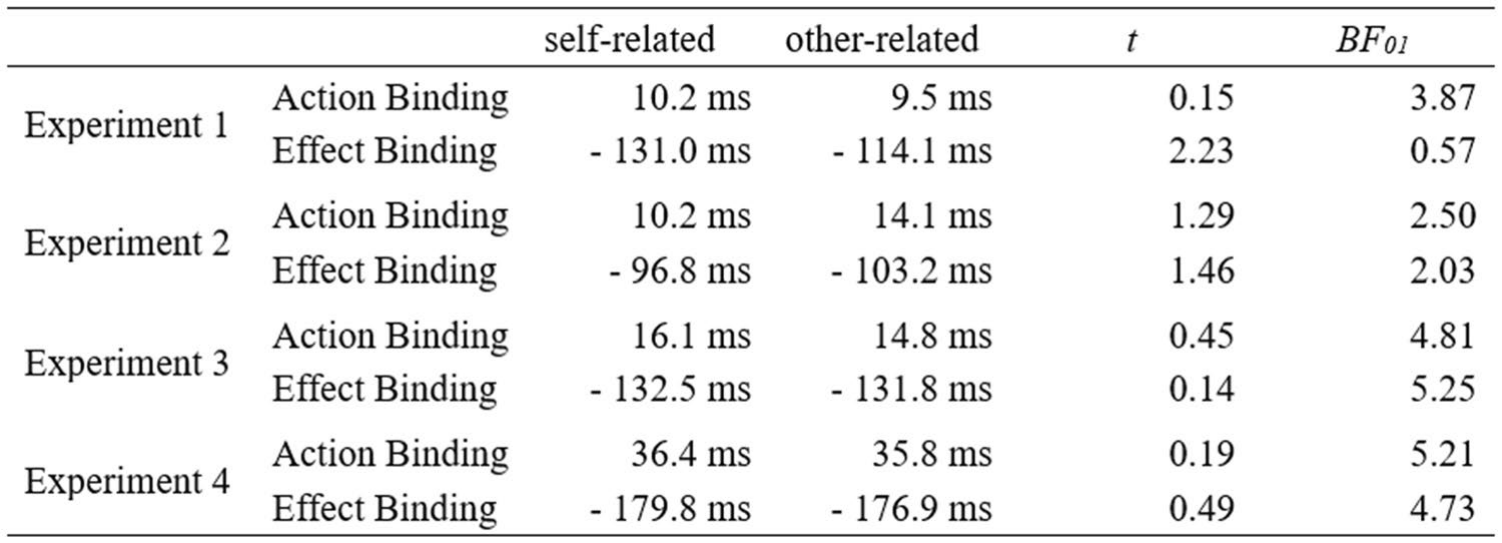
Temporal binding across all four experiments

Action binding and effect binding as mean difference between baseline and operant conditions. Bayes factors were calculated for the difference between self-related and other-related action–effects

### Discussion

Experiment 1 was designed to (a) replicate the results of Makwana and Srinivasan (2019) being that self-related action–effects elicit stronger temporal binding than other-related action–effects. In addition, we intended to locate this effect in either action binding or effect binding or both. Participants performed a temporal binding task in which they either elicited a stimulus which had previously been associated with the self or with another person. In addition, they completed a matching task as manipulation check for the self-relevance manipulation.

Contrary to our expectations, the perceived time points of actions were not shifted to a later point in the operant as compared to the baseline conditions. However, the perceived timing of the outcomes was shifted towards the action in the operant condition compared to the baseline condition. In addition, the difference in effect binding between self-related and other-related action–effects seemed in line with the observation that self-relevance increases temporal binding. However, due to outlier exclusions, our sample size was decreased drastically. A sensitivity analysis revealed that the effect size would have had to be at least 0.80 to be detected with a power of 0.85 in our data indicating that the study might have been underpowered. Moreover, the effect seemed to be smaller than initially expected. In addition, while we found a marginally significant self-prioritization effect in the error rates, reaction times did not differ depending on self-relevance. In addition, error rates in general were quite high which is why the results in the matching task should be interpreted with caution. This indicates that the matching task was rather difficult, and it cannot be concluded that the manipulation check was successful.

Against the backdrop of these observations, we conducted a second experiment with a larger sample size and a slightly easier matching task to replicate and scrutinize the effect of self-relevance on temporal binding and increase the self-prioritization effect.

## Experiment 2: replication—self-relevant shapes

As mentioned above, due to outliers and participant exclusion our sample size in the first experiment shrunk to 16 participants. Thus, we conducted a second experiment aiming at replicating the initial findings in an online study with a larger and more considerate sample size.

Few changes to the initial setup were made (a) to cater for the online setup of the replication study and (b) to strengthen the association between the self and the shape to maximize any effects related to self-relevance. We expected to replicate effect of self-relevance on temporal binding, i.e., to find larger effect binding for self-associated shapes compared to other-associated shapes. Self-relevance was not expected to influence action binding. The study including all changes made to the initial setup as well as participant exclusion criteria was preregistered at the project’s OSF page (https://osf.io/pq43j/).

### Methods

#### Participants

We tested 32 healthy participants recruited over the study platform Prolific (www.prolific.co). They conducted the experiment on their own computer using E-Prime Go (Psychology Software Tools, Inc., 2020). Participants received monetary compensation for their voluntary participation. Based on the medium effect (*d* = 0.56) of self-relevance on effect binding observed in our first experiment, we conducted an a priori sample size calculation for two-tailed paired *t*-tests using G*Power (Faul et al., 2009) with *d* = 0.55, α = 0.05 and a power of 0.85, resulting in a minimal sample size of 32 participants. We had to exclude and replace two participants as they failed to complete more than 33% of the attention checks. The percentage of failed attention checks in the final data set ranged from 0 to 26% (*M* = 7.5, SD = 6.1) Data of one additional participant had to be replaced because some of the data was lost during data collection.^1^ Participants in the final sample set (17 male, 14 female, 1 other; 4 left-handed, 28 right-handed) were between 18 and 47 years (*M* = 27.6, SD = 8.4).

#### Stimuli and task procedure

The experiment consisted of the same four phases as Experiment 1: induction phase, matching phase, agency phase, questionnaires. However, this time we switched the order of the matching phase and the agency phase to strengthen the self-shape association as the self-prioritization effect usually becomes stronger throughout the matching task.

We changed the display time in the agency task from 60 to 100 ms and the size of the geometric shape to fit within the clockface. That is, the square’s edges measured 120 px and the triangle was both 120 px wide and high. In addition, the response window for the self-prioritization task was increased to 1500 ms to decrease the number of misses and errors.

### Results

#### Self-prioritization

Participants did show a self-prioritization effect only in the error rates (see Fig. 2B). Error rates for self-match trials were significantly lower than for other-match trials, *t*(31) = 3.33, *p* = 0.002, *d*_*z*_ = 0.59, BF_10_ = 16.06. Contrary, reactions times did not differ significantly between self-related and other-related trials, *t*(31) = 1.23, *p* = 0.228, *d*_*z*_ = 0.22, ∆ = − 32.2 ms, *BF*_10_ = 0.38. Again, *d’* did not differ significantly between self-related and other-related stimuli, *t*(31) =  − 1.60, *p* = 0.120, *d*_*z*_ = − 0.28.

#### Temporal binding

The 2 × 2 ANOVA for action binding with condition (baseline vs. operant) and relevance (self vs. other) showed that participants tended to report their action to have happened later when it was followed by an outcome than when the event did not occur. However, there is no clear evidence for or against this shift, *F*(1,31) = 3.87, *p* = 0.058, ${\eta }_{p}^{2}$ = 0.11, *BF*_10_ = 2.00. Neither the main effect of relevance nor the interaction were significant, *F*(1,31) = 1.66, *p* = 0.207, ${\eta }_{p}^{2}$ = 0.05.

Both outcomes were perceived to have happened earlier when they were preceded by a keypress as compared to happening in isolation, *F*(1,31) = 39.69, *p* < 0.001, ${\eta }_{p}^{2}$ = 0.56, BF_10_ = 5.02e + 4. The 2 × 2 ANOVA for effect binding did not reveal a main effect of relevance, *F* < 1, or an interaction, *F*(1,31) = 2.13, *p* = 0.155, ${\eta }_{p}^{2}$ = 0.06. That is, there was no significant difference in effect binding between self-related and other-related action–effects.

### Discussion

With Experiment 2, we intended to scrutinize the influence of effect self-relevance of temporal binding. Even though we succeeded in increasing the self-prioritization effect and thereby association strength between the self and the self-relevant stimulus, we could not replicate the effect of outcome relevance on temporal binding. Neither action binding nor effect binding differed significantly between self-related and other-related action–effects.^2^

To draw a preliminary conclusion, a critical reassessment of the changes made to the initial study design is required. First, increasing the response window for the matching task and changing the task order did indeed result in a stronger self-prioritization effect in Experiment 2 compared to Experiment 1. This effect might be strengthened even more when the psychological distance between the self and the other person becomes more pronounced such as the distinction between *self* and *stranger*, which is probably stronger than between *self* and *some other person* (see Golubickis & Macrae, 2021a; Sui et al., 2012). In addition, we had to exclude fewer trials due to too slow reactions. Second, the reduction of the stimulus size to fit within the clock face might have reduced the visual demand in the temporal binding task, however, it did not result in the clear binding pattern we expected. Thus, as the study by Makwana and Srinivasan (2019) used interval estimations and therefore did not have any visual distraction, in our case the clock face, on screen, this additional visual input might have skewed the results. Therefore, we conducted a third experiment with auditory instead of visual action–effects. Thereby, we did not only reduce visual demand but also chose outcomes which have shown to produce stronger and more reliable temporal binding (see Ruess et al., 2018 for a discussion).

## Experiment 3: self-relevant sounds

Following up on the non-significant effects of self-relevance on temporal binding observed in the first two experiments, we conducted another experiment minimizing visual demand in the temporal binding task by using auditory instead of visual action–effects. We were quite confident to be able to replicate the self-prioritization effect as Schäfer et al. (2016) have shown that this effect also translates to the auditory and the tactile domain. The study including all changes made to the other experiments as well as participant exclusion criteria was preregistered at the project’s OSF page (https://osf.io/pq43j/).

### Methods

#### Participants

We tested another set of 32 healthy participants between 20 and 58 years (M = 27.4, SD = 7.8) recruited over the university’s participants pool SONA for in-house data collection. 7 participants identified as male, 25 as female (3 left-handed, 29 right-handed). Participants were naïve regarding the purpose of the study and received monetary compensation for their voluntary participation.

We had to exclude and replace seven participants as they did not correctly remember their sound after completing the matching task. Two additional participants had to be replaced due to a programming error.^3^ In general, participants performed excellently in the attention checks, no participant had to be excluded because they did not identify the encountered stimuli correctly. Missed attention checks ranged between 0 and 15% (*M* = 3.5, SD = 4.1).

#### Stimuli and task procedure

Experiment 3 followed the first two experiments in their setup, however, for time efficiency, in this third experiment we relinquished the questionnaires as the last phase. The order of the three remaining phases was as in the second experiment: induction phase, matching phase, and agency phase.

In contrast to the first two experiments, we used auditory stimuli instead of visual stimuli in Experiment 3 and 4. The two sounds were 300 ms long snippets of a flute and a snare drum. The sounds were neutral in valence (for information on a pilot study see Schäfer et al., 2016) and administered over headphones. At the beginning of the experiment, participants were asked to associate one of two tones (snare drum or flute) with themselves and the other with another person. Each pair was presented six times in the association phase in a random order. Subsequently, participants started the matching task to strengthen the association between the self and the self-associated stimulus. Here, participants decided whether the tone presented matched the simultaneously presented pronoun. Subsequently, in the second part of the experiment, participants performed the temporal binding task in which they could freely choose to press one of two keys producing either the self-related or the other-related tone. The mapping of the keys as well as the self-associated tone was counterbalanced across participants. In addition, participants were asked to press both keys about equally often without following any specific pattern. After every 4th trial, participants indicated the identity of the effect in the trial to ensure that they paid attention to the tone.

### Results

#### Self-prioritization

Participants were faster in correctly identifying self-match compared to other-match trials, *t*(31) = 5.61, *p* < 0.001, *d*_*z*_ = 0.99, ∆ = − 80.4 ms, *BF*_10_ = 5143.26. In addition, they also committed fewer errors in self-match trials compared to other-match trials, *t*(31) = 2.53, *p* = 0.017, *d*_*z*_ = 0.45, BF_10_ = 2.85. The difference in *d’* in favor of other-related stimuli was not significant, *t*(31) =  − 1.97, *p* = 0.058, *d*_*z*_ = − 0.35.

#### Temporal binding

**Fig. 3:**
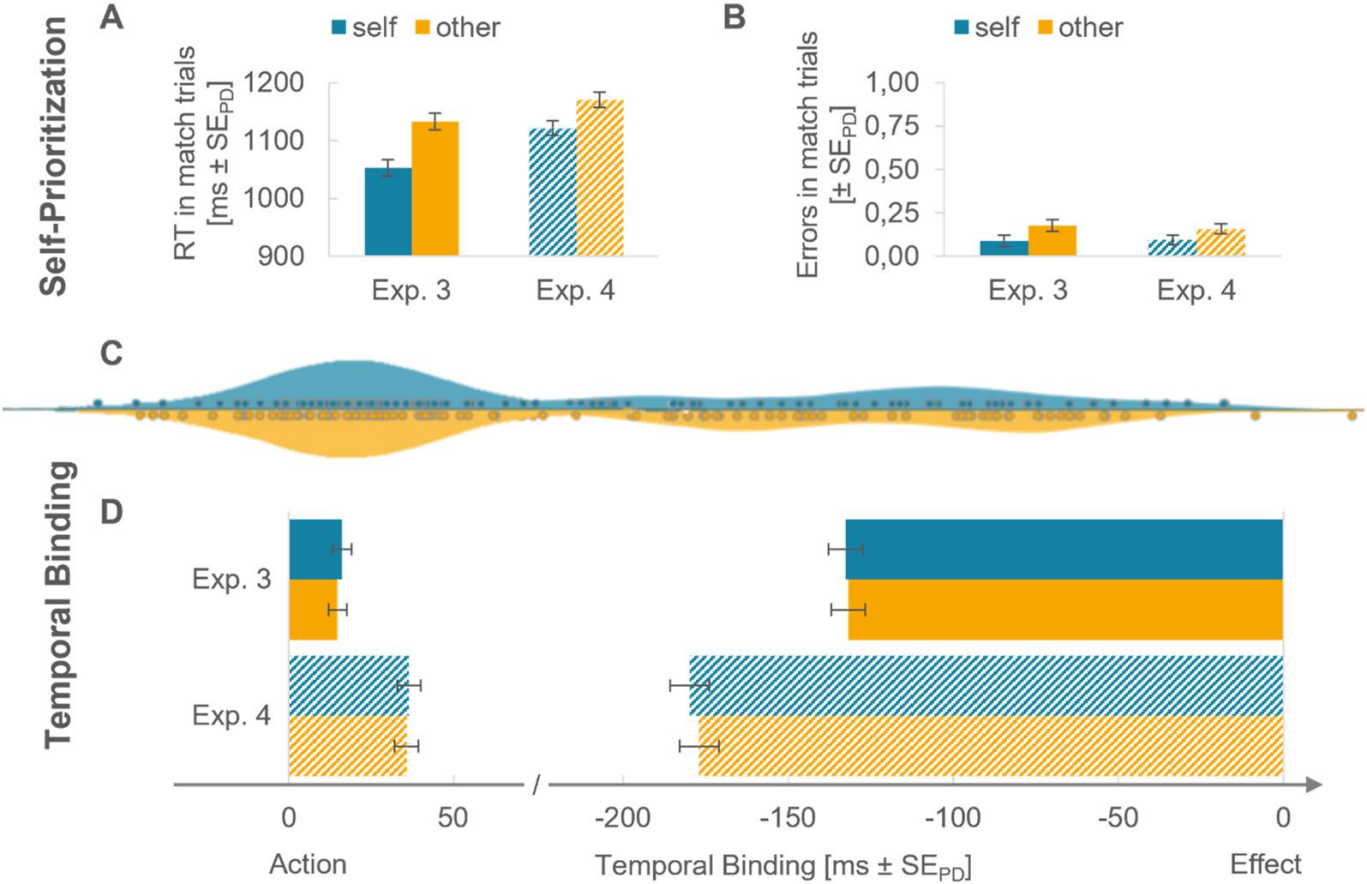
Self-prioritization effect and temporal binding in Experiments 3 and 4 using auditory action–effects. Note. Stimulus association is color-coded: blue is self-related, orange is other-related. **A** Mean reaction times in self-related and other-related matching trials separately for Experiment 3 and Experiment 4. Error bars in all panels depict standard errors for paired differences. **B** Mean error rate in self-related and other-related matching trials separately for Experiment 3 and Experiment 4. **C** Distribution of action binding and effect binding combined for Experiments 3 and 4. Individual points represent participants. **D** Temporal binding in ms separately for Experiment 3 and Experiment 4. Bars from left to right depict the action shift in the operant condition relative to the baseline condition, i.e., action binding. Bars from right to left show the outcome shift in the operant condition relative to the baseline condition, i.e., effect binding

As depicted in Fig. 3D, actions were perceived to have happened later when they were followed by an action–effect as compared to when there was no sound, as indicated by the significant main effect of condition,* F*(1,31) = 7.29, *p* = 0.011, ${\eta }_{p}^{2}$ = 0.19, BF_10_ = 6.12. Neither the main effect of relevance nor the interaction were significant, *Fs* < 1. Similarly, both outcomes were perceived to have happened earlier when they were preceded by a keypress as compared to happening in isolation, *F*(1,31) = 139.40, *p* < 0.001, ${\eta }_{p}^{2}$ = 0.82, BF_10_ = 2.55e + 10. No other comparison was significant, *Fs* < 1. Thus, there was no significant difference in effect binding between self-related and other-related action–effects.

### Discussion

In this third experiment, we used auditory stimuli to analyze any impact of outcome–relevance on temporal binding. Auditory action–effects were used as they were previously found to produce stronger temporal binding compared to visual action–effects (Ruess et al., 2018). In line with our expectations, we did find robust action binding as well as effect binding. However, they did not differ between self-related and other-related outcomes. Again, the manipulation check was successful, and participants exhibited a self-prioritization effect in both reaction times as well as error rates.

## Experiment 4: predictable self-relevance

Finally, to increase the salience of the self-relevance and reduce uncertainty, we conducted a fourth experiment with predictable action–effects. That is, participants’ freely chosen keypresses contingently evoked either the self-related or the other-related outcome. We conducted this experiment to test whether controlling the identity of the upcoming action–effect has an additional influence on the results obtained in the first experiments. However, research on temporal binding and outcome predictability suggests that temporal binding does not differ depending on whether participants have actual control over the outcome identity or whether it is random (Desantis et al., 2012; Haering & Kiesel, 2014). Thus, we expected to replicate the results of Experiment 3. Any effect of outcome relevance here would be due to identity prediction of the outcome.

### Methods

#### Participants

Thirty-two naïve participants of which 12 identified as male and 20 as female were recruited over the study platform Prolific (www.prolific.co) and received monetary compensation for their voluntary participation. Participants (3 left-handed, 28 right-handed, 1 ambidextrous) were between 19 and 38 years old (*M* = 28.0, SD = 5.6). The experiment was conducted on their own computers using E-Prime Go (Psychology Software Tools, Inc., 2020). We conducted Chi-square goodness-of-fit tests to test for equal distribution of key presses and avoid unintended effects of action–effect frequency in the temporal binding task. Four participants were excluded, and their data replaced as they did not meet this criterion. Five subjects were replaced as they did not remember their self-associated sound correctly after the matching task. No additional participant had to be excluded due to failed attention checks which ranged between 0 and 8% (*M* = 1.0, SD = 1.7).

#### Stimuli and task procedure

We used the same stimuli as in Experiment 3 and the procedure followed that of the other experiments. One crucial change in the temporal binding task, however, was that this time, participants could choose between two keys (F and J) that were associated with either of the two outcomes. Both the self-relevant stimulus as well as the mapping of the keys was counterbalanced across participants. They were asked to press each key in about 50% of the trials without following specific patterns. After the first half of each block, participants were informed about the ratio of keypresses so they could adjust in the second half of the block.

### Results

#### Self-prioritization

We found a self-prioritization effect both in the reaction times as well as in the error rates (see Fig. 3A, B). Correct classifications for self-match trials were faster than for other-match trials, *t*(31) = 3.82, *p* < 0.001, *d*_*z*_ = 0.68, ∆ = − 49.1 ms, BF_10_ = 51.04. Similarly, error rates in self-match trials were significantly lower than in other-match trials, *t*(31) = 2.15, *p* = 0.039, *d*_*z*_ = 0.38, *BF*_10_ = 1.42. The sensitivity measure *d’* did not differ between the two types of stimuli, *t*(31) < 1.

#### Temporal binding

Participants judged their actions to have happened later when they were followed by a tone in comparison to when no tone followed, *F*(1,31) = 15.01, *p* < 0.001, ${\eta }_{p}^{2}$ = 0.33, *BF*_10_ = 50.70. The same held true for the outcomes, the perceived timepoints of outcomes was shifted to an earlier time when outcomes were preceded by an action, *F*(1,31) = 91.96, *p* < 0.001, ${\eta }_{p}^{2}$ = 0.75, BF_10_ = 2.14e + 8. There was no significant difference in temporal binding between self-related and other-related action–effects as indicated by the non-significant interaction terms for action binding and effect binding, *Fs* < 1, as well as the Bayes factors that we calculated for the comparisons between self-related outcomes and other-related outcomes for action binding, BF_01_ = 5.21, and effect binding, BF_01_ = 4.73 that both provide strong evidence for no difference.

In addition to the two 2 × 2 ANOVAs for action and effect binding, we conducted paired sample *t*-tests for the estimation errors when the perceived time point of the stimulus had to be judged. Estimation errors did not differ significantly between self-related and other-related outcomes in the baseline condition, *t*(31) = 1.33, *p* = 0.193, *d*_*z*_ = 0.24, ∆ = 4.8 ms, or the operant condition, *t*(31) = 1.67, *p* = 0.106, *d*_*z*_ = 0.29, ∆ = 7.7 ms. This indicates that neither of the sounds had a processing advantage, i.e., was perceived earlier than the other.

#### Pooled analysis

Finally, we conducted pooled analysis including data from all participants reported in the experiments above. Overall, participants were faster at correctly identifying self-match trials compared to other-match trials in the matching task, *t*(111) = 4.84, *p* < 0.001, *d*_*z*_ = 0.46, ∆ = − 47.8 ms. In addition, on average, they also committed fewer errors in self-match trials compared to other-match trials, *t*(111) = 5.13, *p* < 0.001, *d*_*z*_ = 0.49, ∆ = − 9.6%.

The combined evidence for no difference in action binding between self-related and other-related outcomes was strong, BF_01_ = 9.23 (see Figs. 2C and 3C). The same held true for effect binding, BF_01_ = 8.15. This was irrespective of the action–effect modality (visual vs. auditory) and its salience (large vs. small shape). Interestingly, temporal binding appeared to be stronger when participants controlled the action–effect’s identity with their keypresses and therefore were able to predict it (see Fig. 3D). However, post-hoc Dunnett’s test indicated that action and effect binding in the last experiment were only larger than in Experiment 2 (small visual effects) but not in the other two (Table 2).

**Table 2:**
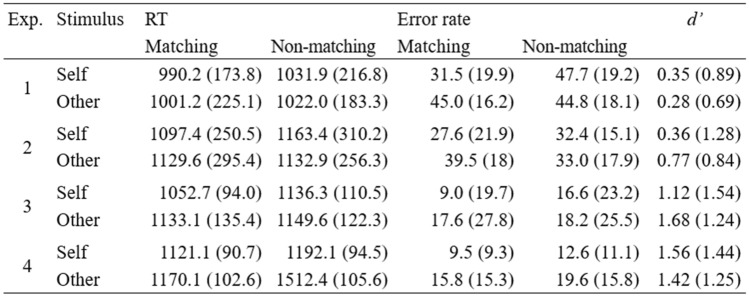
Reaction times, absolute error rates, and the sensitivity measure d’ across all experiments

RTs in milliseconds and absolute error rates in % as well as the sensitivity measure d’ as a function of relevance and matching condition (matching vs. non-matching). Standard deviations are in parentheses

### Discussion

With the fourth experiment we aimed at examining whether the predictability of the outcome’s identity has an additional influence on self-relevance and temporal binding. As predicted, we again did not find an influence of self-relevance on temporal binding. Thus, we conclude that being able to control and predict the outcome’s identity does not moderate the influence of self-relevance on temporal binding.

## General discussion

The present line of research contributes to temporal binding research as well as research on self-prioritization while at the same time bringing the two together. While theorizing as well as preliminary evidence suggest that self-related outcomes produce stronger temporal binding than other-related outcomes, we did not find any influence of self-relevance on temporal binding. In all four experiments, our manipulation checks, i.e., replicating the self-prioritization effect, were reasonably successful. Note, however, that the matching phase was fairly short in comparison to typical self-prioritization studies. We manipulated action–effect modality as well as its salience and its predictability but none of these manipulations proved to have an influence on temporal binding. Nonetheless, we did find significant effect binding in all four individual experiments and action binding in all but the first experiment. We propose two possible mechanisms how self-relevance and temporal binding influence each other. First, self-relevance might only influence temporal binding via immediate response selection. Second, simply being the cause for external events might be sufficient for these events to gain self-relevance.

Research on temporal binding for visual action–effects using the Libet clock is scarce as both time reference and effect are presented in the same modality which might result in reduced salience of action–effects (Moretto et al., 2011; Ruess et al., 2018; but see e.g., Nolden et al., 2012 for visual action–effects and interval estimations). In addition, subjective time perception of visual outcomes could be subject to resolution constrains as the speeds of the pacemakers differ between the visual and auditory domain (Wearden et al., 1998). Our results add to this body of literature by showing that temporal binding for visual action–effects can indeed be measured with the clock method (Experiment 1 and Experiment 2). In addition, the (null-)effects observed for visual stimuli were no different to those we observed with auditory outcomes.

The results presented here seem to contradict those of the original study by Makwana and Srinivasan (2019) and its replication (Chiarella et al., 2020). Reasons for this are manifold and there are a few non-trivial differences in the study design that might account for the diverging results. First, we measured temporal binding with the Libet clock to (a) be able to examine perceived action shifts and action–effect shifts separately and (b) minimize demand effects that might occur when participants retrospectively judge the interval between their action and a specific outcome. Demand characteristics seem to bias interval estimations more easily than the assessment of time perception via the Libet clock. Comparing time estimations between different conditions makes the method more opaque and thus harder to influence. This notion, that interval estimations might be influenced by other high-level processes than time judgements made with the Libet clock, is strengthened by a recent study showing a divergence in these two measures (Siebertz & Jansen, 2022). Consequently, the two measures might be manifestations of different agency experiences where action selection but not action execution is linked to explicit knowledge (see also Hemed et al., 2022; Karsh et al., 2020).

Second, the two previous studies emphasized the self-other reference also during the temporal binding task by asking participants each trial whether the shape they had just produced was associated with the self, a friend, or a stranger. We reduced such influences by simply asking for the identity of the shape (or tone) after every fourth trial and thereby ensured that participants did pay attention to the identity of the action–effect. However, this should not have reduced the strength of the self-relevance manipulation as it facilitates performance as long as a self-relevant dimension, in this case identity of the shape, is part of the task set (Falbén et al., 2019).

Third, the previous studies employed varying delays as an inherent feature of the interval estimation method and the most salient difference between individual identity categories was observed when the outcome was delayed by 400 ms. In the present study, we opted for a constant delay of 250 ms which might not be quite comparable as to the ambiguity it creates for the sensorimotor evaluation of agency. Varying delays may lead to higher ambiguity for sensorimotor evaluation and thus favor high-level cues, in this case stimulus identity, to determine feelings of agency. Consequently, the short delay in the present study would have been an unambiguous (low-level) signal of agency, granting less weight to the high-level cue.

Fourth, while time intervals can technically be inferred from differences in time points, time intervals and time points constitute two perceptually different events. It might well be that certain factors can shape the perception of time intervals, as used by Makwana and Srinivasan (2019), but not time points, as used in the present study. Future research should consider this by varying the delays between action and outcome, and by reading out different perceptual aspects of the same physical events from the same participants.

Finally, in the present study, participants performed the temporal binding task as well as the matching task in one session, whereas Makwana and Srinivasan (2019) invited participants to the lab twice—once for a longer matching session and once to complete a short matching block followed by the interval estimation task. While this elongated period might have strengthened the association between the self and the arbitrary stimulus, data of our matching task clearly showed, that participants were able to pick up a strong association in the time provided. Consequently, we conjecture that the varying levels of induction of self-relevance are a less likely explanation of the diverging result patterns rather than other possible causes such as weaker demand effects in the current temporal binding measure than in the interval estimation procedure used in previous studies.

As we could not replicate previous findings, the question must be raised whether there is an effect of self-relevance on temporal binding at all. As of yet, there is no clear answer to this question, but we propose two arguments to explain the lack of influence of self-relevance on temporal binding in the present study. This opens new perspectives for future research in the field.

First, stimulus processing and response selection possibly moderate the influence of self-relevance on temporal binding. Initially, Humphreys and Sui (2016) argued that the self-prioritization effect stems from an early processing bias in attentional control towards self-related information. However, Schäfer et al. (2020) could show that other information such as negative valence can derail attention at an earlier stage indicating that self-related information does not trump mere perceptual input. In line with this, the lack of stronger temporal binding of self-relevant action–effects suggests that self-related stimuli are not processed faster perceptually, compared to other-related stimuli. That is, the estimation errors for both effect-occurrences (self-related and other-related) were equal, even though participants reacted faster and more accurately to self-match trials compared to other-match trials in the matching task. This suggests that the self-prioritization effect does not reflect perceptual benefits of self-related stimuli, i.e., earlier perception, but rather advantages in later/other processing stages such as response selection or response execution, in case such selection is required. Consequently, the expected modulation might occur if the identity of the outcome is required to generate an appropriate motor response to this outcome indicating prioritization in the anticipation of self-relevant action–effects (e.g., Kunde, 2001; Pfister et al., 2010). This idea is supported by Woźniak and Knoblich (2021) who suggest that the self-association has to be active in working memory to elicit a self-prioritization effect Additional work indicates that the automaticity of self-prioritization is conditional to attention on the self-relevance of the object to be classified (Caughey et al., 2021; Falbén et al., 2019). Neither previous studies nor the current study design allow to test this mechanism. Hence, to further resolve the puzzle whether self-relevance influences temporal binding, future research could interlace the temporal binding and self-prioritization task in such a way that temporal binding is measured in combination with continuous speeded responses.

Second, causing external events might suffice for these to become self-relevant. The specific setup of the current studies caters to this explanation. Knowing that an event is going to occur and even being able to predict its nature and timing helps the organism to prepare for this specific event. Thus, it will be expected and carry relevance. In contrast to the matching task, where the stimulus-label combination serves as symbol to trigger an action that must be retrieved from memory, in the temporal binding task, the stimulus serves as action–effect. Here, participants retrospectively have to retrieve the timing as well as the identity (for unpredictable outcomes) of the perceived sensory input to make judgements about its occurrence (see Moore & Haggard, 2008; Reddy, 2022). Attention focusses more quickly on self-relevant stimuli (see also Golubickis & Macrae, 2021b) making them accessible earlier to the system whereby effect binding should increase. Yet, such speeded attentional focusing might only occur with immediate action planning but not with retrospective judgements of sensory events. In the same vein, Golubickis et al. (2017) found temporal influences on the self-prioritization effect such that only stimuli associated with the current self, as compared to a future or past self, facilitated reaction times and accuracy indicating that the attentional benefit of self-relevant information is timely limited. Knowing whether a sensory event in the outside world was caused by oneself or not is crucial for human learning and development throughout all stages of life (Engbert & Wohlschläger, 2007; Kunde et al., 2018; Schaaf et al., 2022). Thus, an agent’s knowledge of their effectiveness in causing a certain outcome might be enough for this specific event to gain self-relevance. In consequence, stimuli which have previously been associated with someone else become self-relevant, too, just by the fact that they were caused by an own motor action. Hence, the lack of influence of the outcome’s self-relevance on temporal binding. One possibility to address this would be to reduce participants’ effectiveness, e.g., by introducing longer action–outcome delays, by varying action–outcome contingency, or by increasing causal uncertainty through other agents. In these cases, the outcome’s self-relevance provides additional information about the agent’s efficiency and might thus facilitate temporal binding.

## Conclusion

We conducted four experiments to analyze influences of outcome self-relevance on temporal binding. While participants exhibited a robust self-prioritization effect in all four experiments, we only found anecdotal evidence for a modulation of temporal binding through the self-relevance of action–effects in the first experiment. All other experiments as well as the pooled analyses provided strong evidence for no effect of outcome self-relevance on temporal binding. In addition, estimation errors did not differ between self-related and other-related stimuli. Thus, we conclude that possible attentional shifts responsible for self-prioritization might occur when response selection regarding these stimuli is required, e.g., in continuous speeded response tasks, while it does not occur when indicating the onset of an action–effect irrespective of its identity, as in temporal binding. Alternatively, merely causing any outcome in the environment might be sufficient for this event to become self-relevant irrespective of the previously formed self-association.

### Supplementary Information

Below is the link to the electronic supplementary material.

Supplementary file1 (DOCX 16 KB)

## Data Availability

All experiments were preregistered on the Open Science Framework (OSF) and raw data as well as additional materials are available (https://osf.io/pq43j/).

## Footnotes

1 Data of all participants including replaced subjects is available at the project’s OSF repository.

2 We conducted an additional experiment as replication of Experiment 1 with the same task order to control for possible task sequence effects. Here, we did not find any modulation of temporal binding, either. Data and analysis are available at the project’s OSF page (https://osf.io/pq43j/).

3 Again, data of these participants is available at the project’s OSF repository.

## Code availability

All experiments were preregistered on the Open Science Framework (OSF) and raw data as well as additional materials are available (https://osf.io/pq43j/).

## References

[CR1] Bartlett, J. (2017). *An introduction to JASP: A free and user-friendly statistics package*. https://osf.io/p2hzg.

[CR2] Beck B, Di Costa S, Haggard P (2017). Having control over the external world increases the implicit sense of agency. Cognition.

[CR3] Brailovskaia J, Bierhoff H-W, Margraf J (2019). How to identify narcissism with 13 items? Validation of the German Narcissistic Personality Inventory–13 (G-NPI-13). Assessment.

[CR4] Buehner MJ (2012). Understanding the past, predicting the future: Causation, not intentional action, is the root of temporal binding. Psychological Science.

[CR5] Buehner MJ, Humphreys GR (2009). Causal binding of actions to their effects. Psychological Science.

[CR6] Caspar EA, Cleeremans A, Haggard P (2018). Only giving orders? An experimental study of the sense of agency when giving or receiving commands. PLoS ONE.

[CR7] Caughey S, Falbén JK, Tsamadi D, Persson LM, Golubickis M, Neil Macrae C (2021). Self-prioritization during stimulus processing is not obligatory. Psychological Research Psychologische Forschung.

[CR8] Chiarella SG, Makwana M, Simione L, Hartkamp M, Calabrese L, Raffone A, Srinivasan N (2020). Mindfulness meditation weakens attachment to self: Evidence from a self vs other binding task. Mindfulness.

[CR9] Christensen JF, Yoshie M, Di Costa S, Haggard P (2016). Emotional valence, sense of agency and responsibility: A study using intentional binding. Consciousness and Cognition.

[CR10] Constable MD, Kritikos A, Bayliss AP (2011). Grasping the concept of personal property. Cognition.

[CR11] Constable MD, Welsh TN, Huffman G, Pratt J (2019). I before U: Temporal order judgements reveal bias for self-owned objects. Quarterly Journal of Experimental Psychology.

[CR12] Cunningham SJ, Turk DJ, Macdonald LM, Neil Macrae C (2008). Yours or mine? Ownership and memory. Consciousness and Cognition.

[CR13] Desantis A, Hughes G, Waszak F (2012). Intentional binding is driven by the mere presence of an action and not by motor prediction. PLoS ONE.

[CR14] Desebrock C, Spence C (2021). The self-prioritization effect: Self-referential processing in movement highlights modulation at multiple stages. Attention, Perception, & Psychophysics.

[CR15] Desebrock C, Sui J, Spence C (2018). Self-reference in action: Arm-movement responses are enhanced in perceptual matching. Acta Psychologica.

[CR16] Dienes Z (2014). Using Bayes to get the most out of non-significant results. Frontiers in Psychology.

[CR17] Dienes Z (2019). How do I know what my theory predicts?. Advances in Methods and Practices in Psychological Science.

[CR18] Engbert K, Wohlschläger A (2007). Intentions and expectations in temporal binding. Consciousness and Cognition.

[CR19] Falbén JK, Golubickis M, Balseryte R, Persson LM, Tsamadi D, Caughey S, Neil Macrae C (2019). How prioritized is self-prioritization during stimulus processing?. Visual Cognition.

[CR20] Faul F, Erdfelder E, Buchner A, Lang A-G (2009). Statistical power analyses using G*Power 3.1: Tests for correlation and regression analyses. Behavior Research Methods.

[CR21] Frings C, Wentura D (2014). Self-priorization processes in action and perception. Journal of Experimental Psychology: Human Perception and Performance.

[CR22] Golubickis M, Falbén JK, Sahraie A, Visokomogilski A, Cunningham WA, Sui J, Macrae CN (2017). Self-prioritization and perceptual matching: The effects of temporal construal. Memory & Cognition.

[CR23] Golubickis M, Ho NSP, Falbén JK, Schwertel CL, Maiuri A, Dublas D, Cunningham WA, Macrae CN (2021). Valence and ownership: Object desirability influences self-prioritization. Psychological Research Psychologische Forschung.

[CR24] Golubickis M, Macrae CN (2021). Judging me and you: Task design modulates self-prioritization. Acta Psychologica.

[CR25] Golubickis M, Macrae CN (2021). That’s me in the spotlight: Self-relevance modulates attentional breadth. Psychonomic Bulletin & Review.

[CR26] Gräfe K, Zipfel S, Herzog W, Löwe B (2004). Screening psychischer Störungen mit dem “Gesundheitsfragebogen für Patienten (PHQ-D)”. Diagnostica.

[CR27] Haering C, Kiesel A (2014). Intentional binding is independent of the validity of the action effect’s identity. Acta Psychologica.

[CR28] Haggard P, Clark S (2003). Intentional action: Conscious experience and neural prediction. Consciousness and Cognition.

[CR29] Haggard P, Clark S, Kalogeras J (2002). Voluntary action and conscious awareness. Nature Neuroscience.

[CR30] Haggard P, Tsakiris M (2009). The experience of agency. Current Directions in Psychological Science.

[CR31] Hemed E, Karsh N, Mark-Tavger I, Eitam B (2022). Motivation (s) from control: Response-effect contingency and confirmation of sensorimotor predictions reinforce different levels of selection. Experimental Brain Research.

[CR32] Hoerl C, Lorimer S, McCormack T, Lagnado DA, Blakey E, Tecwyn EC, Buehner MJ (2020). Temporal binding, causation, and agency: Developing a new theoretical framework. Cognitive Science.

[CR33] Hon N (2023). Attention and expectation likely underlie temporal binding measured using the Libet clock. Quarterly Journal of Experimental Psychology.

[CR34] Humphreys GW, Sui J (2016). Attentional control and the self: The Self-Attention Network (SAN). Cognitive Neuroscience.

[CR35] JASP Team. (2018). *JASP [Computer software]* (Version 0.8. 5.1) [Computer software]. https://jasp-stats.org/.

[CR36] Kahneman D, Knetsch JL, Thaler RH (1991). Anomalies: the endowment effect, loss aversion, and status quo bias. Journal of Economic Perspectives.

[CR37] Karsh N, Hemed E, Nafcha O, Elkayam SB, Custers R, Eitam B (2020). The differential impact of a response’s effectiveness and its monetary value on response-selection. Scientific Reports.

[CR38] Kirsch W, Kunde W, Herbort O (2019). Intentional binding is unrelated to action intention. Journal of Experimental Psychology: Human Perception and Performance.

[CR39] Klaffehn AL, Sellmann FB, Kirsch W, Kunde W, Pfister R (2021). Temporal binding as multisensory integration: Manipulating perceptual certainty of actions and their effects. Attention, Perception, & Psychophysics.

[CR40] Kunde W (2001). Response-effect compatibility in manual choice reaction tasks. Journal of Experimental Psychology: Human Perception and Performance.

[CR41] Kunde W, Weller L, Pfister R (2018). Sociomotor action control. Psychonomic Bulletin & Review.

[CR42] Lush P, Roseboom W, Cleeremans A, Scott RB, Seth AK, Dienes Z (2019). Intentional binding as Bayesian cue combination: Testing predictions with trait individual differences. Journal of Experimental Psychology: Human Perception and Performance.

[CR43] Makwana M, Srinivasan N (2019). Self-associated stimuli produce stronger intentional binding. Journal of Experimental Psychology: Human Perception and Performance.

[CR44] Moore JW, Haggard P (2008). Awareness of action: Inference and prediction. Consciousness and Cognition.

[CR45] Moore JW, Obhi SS (2012). Intentional binding and the sense of agency: A review. Consciousness and Cognition.

[CR46] Moreton J, Callan MJ, Hughes G (2017). How much does emotional valence of action outcomes affect temporal binding?. Consciousness and Cognition.

[CR47] Moretto G, Walsh E, Haggard P (2011). Experience of agency and sense of responsibility. Consciousness and Cognition.

[CR48] Nolden S, Haering C, Kiesel A (2012). Assessing intentional binding with the method of constant stimuli. Consciousness and Cognition.

[CR49] Pfister R, Kiesel A, Melcher T (2010). Adaptive control of ideomotor effect anticipations. Acta Psychologica.

[CR50] Pfister R, Obhi SS, Rieger M, Wenke D (2014). Action and perception in social contexts: Intentional binding for social action effects. Frontiers in Human Neuroscience.

[CR51] Psychology Software Tools, Inc. (2020). *E-Prime Go 1.0* [Computer software]. Pittsburgh, PA. https://support.pstnet.com/.

[CR52] Reddy NN (2022). The implicit sense of agency is not a perceptual effect but is a judgment effect. Cognitive Processing.

[CR53] Ruess M, Thomaschke R, Kiesel A (2017). The time course of intentional binding. Attention, Perception, & Psychophysics.

[CR54] Ruess M, Thomaschke R, Kiesel A (2018). Intentional binding of visual effects. Attention, Perception, & Psychophysics.

[CR55] Schaaf M, Kunde W, Wirth R (2022). Evidence for initially independent monitoring of responses and response effects. Journal of Experimental Psychology: Human Perception and Performance.

[CR56] Schäfer S, Wentura D, Frings C (2015). Self-prioritization beyond perception. Experimental Psychology.

[CR57] Schäfer S, Wentura D, Frings C (2020). Creating a network of importance: The particular effects of self-relevance on stimulus processing. Attention, Perception, & Psychophysics.

[CR58] Schäfer S, Wesslein A-K, Spence C, Frings C (2021). When self-prioritization crosses the senses: Crossmodal self-prioritization demonstrated between vision and touch. British Journal of Psychology (london, England).

[CR59] Schäfer S, Wesslein A-K, Spence C, Wentura D, Frings C (2016). Self-prioritization in vision, audition, and touch. Experimental Brain Research.

[CR60] Siebertz M, Jansen P (2022). Diverging implicit measurement of sense of agency using interval estimation and Libet clock. Consciousness and Cognition.

[CR61] Silver CA, Tatler BW, Chakravarthi R, Timmermans B (2021). Social agency as a continuum. Psychonomic Bulletin & Review.

[CR62] Sui J, He X, Humphreys GW (2012). Perceptual effects of social salience: Evidence from self-prioritization effects on perceptual matching. Journal of Experimental Psychology: Human Perception and Performance.

[CR63] Sui J, Humphreys GW (2015). The interaction between self-bias and reward: Evidence for common and distinct processes. Quarterly Journal of Experimental Psychology.

[CR64] Sui J, Rotshtein P, Humphreys GW (2013). Coupling social attention to the self forms a network for personal significance. Proceedings of the National Academy of Sciences of the United States of America.

[CR65] Takahata K, Takahashi H, Maeda T, Umeda S, Suhara T, Mimura M, Kato M (2012). It’s not my fault: Postdictive modulation of intentional binding by monetary gains and losses. PLoS ONE.

[CR66] Thanopoulos V, Psarou E, Vatakis A (2018). Robust intentional binding for causally-linked sequences of naturalistic events but not for abstract event sequences. Acta Psychologica.

[CR67] Tonn S, Pfister R, Klaffehn AL, Weller L, Schwarz KA (2021). Two faces of temporal binding: Action-and effect-binding are not correlated. Consciousness and Cognition.

[CR68] Tsakiris M, Haggard P (2005). Experimenting with the acting self. Cognitive Neuropsychology.

[CR69] Wearden JH, Edwards H, Fakhri M, Percival A (1998). Why “sounds are judged longer than lights”: Application of a model of the internal clock in humans. The Quarterly Journal of Experimental Psychology: Section B.

[CR70] Wegner DM (2002). The Illusion of Conscious Will.

[CR71] Wolpe N, Haggard P, Siebner HR, Rowe JB (2013). Cue integration and the perception of action in intentional binding. Experimental Brain Research.

[CR72] Woźniak M, Knoblich G (2021). Self-prioritization depends on assumed task-relevance of self-association. Psychological Research Psychologische Forschung.

[CR73] Yamamoto K (2020). Cue integration as a common mechanism for action and outcome bindings. Cognition.

