# Supplementary material for: You do you: susceptibility of temporal binding to self-relevance
Source: Psychol Res. 2024 Jan 3;88(3):1007–22. doi: 10.1007/s00426-023-01906-9 (PMC10965574; doi:10.1007/s00426-023-01906-9)
Supplement: Supplementary file 1 — Supplementary file1 (DOCX 16 KB) [file 426_2023_1906_MOESM1_ESM.docx]

# **Supplement**

Table 3

*2 × 2 repeated measures ANOVAs for RTs and % correct in the matching task*

|  |  |  | RT |  |  | % correct |  |
| --- | --- | --- | --- | --- | --- | --- | --- |
| Experiment |  | *F* | *p* | *ηp2* | *F* | *p* | *ηp2* |
| 1 | Pronoun | 0.51 | .486 | .03 | 5.04 | .040 | .52 |
|  | Trial type | 3.91 | .067 | .21 | 2.84 | .113 | .16 |
|  | Interaction | 0.01 | .944 | .01 | 2.08 | .170 | .12 |
| 2 | Pronoun | 3.44 | .073 | .10 | 5.63 | .024 | .15 |
|  | Trial type | 5.46 | .026 | .15 | 0.10 | .752 | .01 |
|  | Interaction | 0.01 | .962 | .01 | 9.13 | .005 | .23 |
| 3 | Pronoun | 15.06 | .001 | .33 | 4.09 | .052 | .12 |
|  | Trial type | 38.59 | .001 | .56 | 6.00 | .020 | .16 |
|  | Interaction | 26.96 | .001 | .47 | 3.78 | .061 | .11 |
| 4 | Pronoun | 2.99 | .094 | .09 | 0.07 | .790 | .01 |
|  | Trial type | 46.85 | .001 | .60 | 7.23 | .011 | .19 |
|  | Interaction | 12.04 | .002 | .28 | 8.57 | .006 | .22 |

*Note.* Pronoun (self vs. other) and trial type (match vs. no-match) as within-subject factors.

In Experiment 1, we could not replicate typical findings of the self-prioritization effect, i.e., faster RTs for match compared to no-match trials, which raises concern about whether the task setup was suitable to elicit a self-prioritization effect. However, in all other three experiments, match trials were faster than no-match trials (main effect of trial type), replicating typical results from previous studies on self-prioritization.
